# Characterizing Information Propagation in Social Media with Branching Processes

**DOI:** 10.3390/e28050493

**Published:** 2026-04-26

**Authors:** Xiaofang Luo, Haibo Hu, Qingsong Sun

**Affiliations:** 1 Department of Management Science and Engineering, East China University of Science and Technology, Shanghai 200237, China; 2 School of Future Technology, Anhui Finance and Trade Vocational College, Hefei 230601, China

**Keywords:** information propagation, branching process, social media, data-driven modeling

## Abstract

Information propagation in social media has attracted the wide attention of scholars, with great progress made in empirical and modeling studies. Branching processes, extensively utilized in theoretical biology, are increasingly applied to model information diffusion dynamics. However, detailed and data-driven studies that implement this methodology remain rare. This study, utilizing empirical data, characterizes and models information diffusion in social media with branching processes. The reliability of the branching model is verified through the comparison of theoretical predictions, numerical simulations, and empirical results, and the model can replicate the key statistical characteristics observed in realistic cascades. The research results validate the applicability of branching processes in information diffusion, and contribute to the development of more elaborate, data-driven models of information spreading in complex real-world scenarios.

## 1. Introduction

Social media platforms, such as X/Twitter, Facebook, and Weibo, play a crucial role in people’s daily interactions, significantly altering the way of information production, diffusion and consumption on the internet. Individuals are not only recipients of information but also actively participate in information spreading through forwarding and commenting, exerting a huge influence on public opinions, consumer behaviors and public health.

Information diffusion in social media has attracted the wide attention of scholars from diverse disciplines, and significant progress has been made in empirical and modeling research [1,2,3,4,5,6,7]. In this field, early research focused on monolayer networks, and in recent years, information spreading on multilayer or higher-order networks has also been explored [8,9,10,11].

Empirical research reveals the spreading mechanisms [12,13], meme popularity [14,15], and temporal patterns [15,16] of information diffusion. The majority of cascades go extinct in the early stage, while a few show explosive growth, and the size and duration of cascades follow power-law or heavy-tailed distributions [14,15]. Even for similar-scale cascades, the diffusion trees exhibit high structural diversity, and the content popularity has little correlation with the structural virality [17]. Large-scale dissemination is often driven by a few key nodes [17]. Moreover, the analysis on propagation paths indicates that, while social networks display pronounced clustering and complex topology, individual information diffusion cascades often exhibit a tree-like structure locally [18], which offers an empirical foundation for modeling information dissemination—for instance, through a branching process.

Regarding spreading models, percolation theory, compartmental models, branching processes and Hawkes processes have been employed to investigate the information diffusion dynamics [19]. Percolation theory models the emergence of system-wide propagation [5], epidemic-like compartmental models (SIS, SIR, etc.) model spreading dynamics by concentrating on individual state transition [20,21], branching processes model reproduction dynamics generation by generation [22,23], and Hawkes processes model the realistic temporal patterns of spreading events [24,25,26]. These models are not isolated; rather, they are closely linked. Compartmental models [27,28,29,30], branching processes [31] and Hawkes processes [16] are also utilized to study the competitive information diffusion or information interaction.

The theoretical models reveal the influence of the information dissemination mechanism and network topology on the diffusion threshold, scope and speed [32,33,34,35], while data-driven models explain and generate the observed spreading patterns by integrating the propagation models with empirical data. Based on online diffusion, a model of contagion derived from the SIR model with a low infection rate was proposed. Although several empirical findings are consistent with the model, it fails to replicate the diversity of structural virality in real-world diffusion [17]. In social networks, diffusion records show that diffusion probability along a social tie follows a power-law relationship with the number of the disseminator’s followers and the receiver’s followees. A cascade model incorporating this finding was proposed that can reproduce key structural features of diffusion trees [4]. Utilizing Weibo and Twitter data, the data-driven percolation model was also proposed to reproduce the cascade size and threshold [5]. During information spreading, it was found that both social reinforcement and weakening effects coexist. The forwarding probability of information initially increases with exposure frequency, then subsequently plateaus or decreases. A model considering the feature can describe the empirical spreading trajectories well [7]. Some data-driven models connect multiple frameworks. For example, the SIR model can be integrated with a Hawkes process in which the rate of events in the latter is identical to the rate of new infections in the former [24]. The bimodal distribution of cascade sizes from the model may provide explanations to the general unpredictability of popularity.

Among the diffusion models, the branching process (Bienaymé–Galton–Watson process) was initially developed to investigate the potential extinction of family surnames resulting from insufficient descendant numbers, and can model the information spreading over successive generations. Branching processes can reproduce realistic diffusion characteristics. For instance, the diffusion of internet chain letters shows the “long-chain, narrow-tree” structure that can be explained by incorporating the Galton–Watson process with selection bias [36]. The viral marketing information spreading displays non-Markovian branching dynamics that can be modeled by a two-step Bellman–Harris branching process that incorporates the high variability of user behaviors [22]. Analysis on information cascades on Twitter reveals that branching processes can characterize the spreading process, reproducing the statistical characteristics of the empirical cascadevs [23].

In social media, meme popularity and cascade duration can follow power-law distributions with diverse scaling exponents, and branching processes can explain the distribution well [14,15,31]. Indeed, due to the inherent complexity involved in the information dissemination, branching processes have limited applicability across diverse real-world scenarios. It was revealed that branching processes have stronger explanatory power when depicting simple contagion or small-scale cascades, while a random-field Ising model can better describe complex contagion [15]. Branching processes can be used to predict the diffusion reach [37] and reveal influential nodes [38], and this approach has also been further applied to issues such as anomaly detection and misinformation modeling. For instance, a statistical testing framework based on a branching process was proposed to distinguish normal branches from abnormal cascades in networks, and this method could detect key violent events during the 2011 Yemeni Revolution [39]. A branching process was also utilized to model the spread of misinformation in both passive and active environments, and structural metrics such as propagation probability, height, and size can be analytically studied [40].

Further, multitype branching processes have also been utilized to study information diffusion [41]. For instance, the SIR model and a multitype branching process were used to analyze the information spreading in two-layer clustered networks; the epidemic threshold and the final outbreak size can be derived, and the network clustering can inhibit the spread [42]. However, a study on seed-initiated cascades on clustered networks with a two-type branching process revealed that for a low average degree, clustering impedes cascade propagation, but for a high average degree, clustering promotes the spread [43]. By constructing a multitype branching process, complex contagion on clustered networks [44,45] and simple contagion on networks with communities [46] were also analyzed, and the cascade size and critical behavior were analytically studied. Some scholars introduced temporal variables, and through a timeline-based multitype branching process, derived the number of post shares and the probability of virality or extinction [47]. Multitype branching processes can be used to study spreading processes across multilayer networks [48,49], yielding a universal meme popularity distribution characterized by a robust power-law exponent [15,48]. They were also employed to study the containment of fake news and misinformation [50,51].

Although information dissemination and, more specifically, branching processes have received considerable attention, several gaps remain in current research. First, existing studies often focus on critical thresholds and diffusion scales, but rarely explore, both empirically and theoretically, how microscopic diffusion behaviors generate macroscopic diffusion patterns. Second, in data-driven studies, the models are often coarsely granulated, and model parameters are seldom calibrated against empirical data. Third, theoretical models frequently lack rigorous integration with real-world data sets, thereby limiting their explanatory validity.

To address these gaps, utilizing empirical data, this paper characterizes and models information diffusion in social media with branching processes. Inspired by the work of Gleeson et al. [23], this study examines the effective branching numbers and offspring distributions in real-world data, and validates the applicability of branching processes to the data under study. The reliability of the branching model is verified by comparing the theoretical predictions, numerical simulations, and empirical results.

The remainder of this paper is organized as follows. Section 2 presents the data sets under study. Section 3 undertakes empirical analysis and parameter estimation. Section 4 employs the branching process model to characterize diffusion dynamics and replicates key propagation patterns observed in data. Section 5 discusses several special cases. Section 6 concludes the paper and outlines promising avenues for future research.

## 2. Data Sets

This paper conducts empirical analysis and modeling study using three data sets from Weibo (Chinese version of X/Twitter) whose fundamental characteristics are summarized in Table 1. Weibo is the largest microblogging platform in China, and the three data sets are each large-scale and cover different topics. These data sets were collected in the same year, and the events covered by them went through the entire process from occurrence to conclusion within that year.

The first data set comprises posts related to the TV talent show “Produce 101” in China. “Produce 101” is a multi-stage elimination competition featuring 101 contestants undergoing multiple rounds of assessment and public voting. The show was delivered via online live streaming, and the audience can generate and disseminate content related to the show on Weibo. The time span of the data set, i.e., from 1 April to 30 June 2018, covers the full lifecycle of the show from pre-launch promotion and contestant training through to the finale. The second data set comprises posts related to a self-defense case in Kunshan, Jiangsu Province, China. The incident originated from a traffic-related dispute between a car driver and a cyclist. During the altercation, the driver took out a knife from the car and continuously assaulted the cyclist with it. In response, the cyclist snatched the knife back and stabbed and slashed the driver several times. The driver sustained critical injuries and succumbed to them despite immediate medical intervention. The incident attracted widespread public and legal attention. The data set, with a time span from 27 August to 2 September 2018, covers the entire course of the incident—from its initial public disclosure and subsequent media coverage, through official investigations, to the formal announcement of the decision by the Kunshan Municipal Public Security Bureau.

The third data set comprises posts related to the Chinese film “Dying to Survive”. The medically themed film told the story of a grassroots health product salesman who, facing financial hardship and mounting rent pressures, became the exclusive selling agent of unapproved generic versions of life-saving anticancer drugs from India to meet urgent patient needs. The data set, with a time span from 1 May to 7 August 2018, includes the entire cycle from the film’s promotion, release to its gradual withdrawal.

All three data sets contain the complete forwarding chains of original posts. We construct information diffusion trees based on the observed repost (forwarding) relationships in Weibo. For each original post, all posts directly or indirectly derived from it through forwarding collectively constitute a diffusion tree—representing the complete propagation pathway of that original post. The tree can be regarded as a directed network, where nodes are posts, and the directed edges represent the forwarding relationship between posts or the forwarding behavior of users. In the network, the node with zero in-degree is the seed node or root node (i.e., the original post), nodes with zero out-degree are leaf nodes, and nodes possessing both non-zero in-degree and out-degree are viral nodes.

## 3. Empirical Analyses

### 3.1. Effective Branching Numbers

We employ the method outlined in the work of Gleeson et al. to analyze three social media data sets [23]. In the branching process, for a diffusion tree, let $Z_{n}$ (n≥0) be the size (i.e., number of nodes) of the *n*th generation. When n=0, $Z_{n}$ denotes the number of root nodes (usually, $Z_{0}=1$ for a diffusion tree). The effective branching number of the *n*th generation, denoted by $\omega_{n}$, is defined as the ratio of the size of the (*n* + 1)th generation to that of the *n*th generation, i.e., $\omega_{n}=Z_{n+1}/Z_{n}$. For a data set, since there are a large number of diffusion trees, $\omega_{n}$ is defined as the ratio of the total number of nodes in the (*n* + 1)th generation to that in the *n*th generation for all diffusion trees [23].

**Figure 1:**
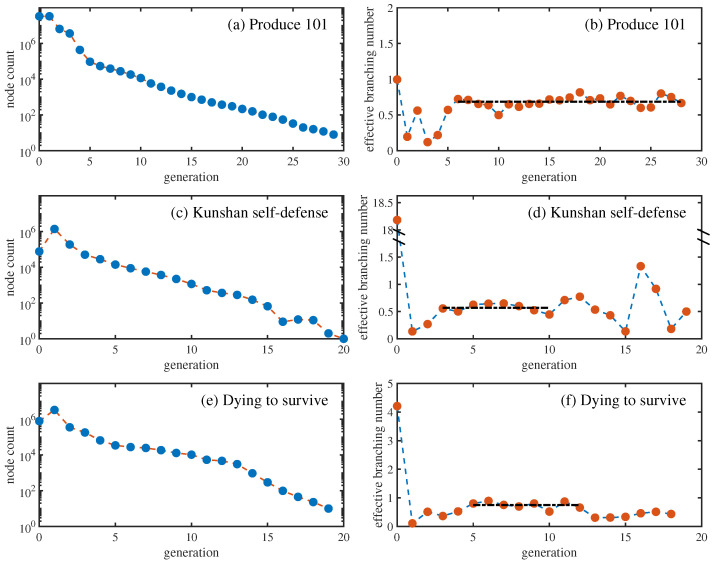
The total node numbers (**a**,**c**,**e**) and the effective branching numbers (**b**,**d**,**f**) across generations for the three data sets. For the generations with stable effective branching numbers, the corresponding average values are indicated by dot-dashed lines.

Figure 1 displays the total numbers of nodes and the effective branching numbers across generations for the three data sets. It can be observed that as the generation number increases, the node number generally decreases gradually. On the log-linear axes, this decline approximates a straight line, suggesting exponential decay. Notably, “Produce 101” displays pronounced fluctuations in node count only at early generations, whereas “Kunshan self-defense” and “Dying to survive” exhibit obvious variability at both early and late generations.

As for the effective branching number, it can be seen that for “Produce 101”, between the 6th and 28th generation, the effective branching number tends to stabilize as the generation increases, with an average of 0.685; for “Kunshan self-defense”, stability is observed between the 3rd and 10th generation, yielding a mean of 0.568; and for “Dying to survive”, reduced fluctuations and relative stability occur from the 5th to 12th generation, with a mean of 0.749. The basic reproductive number $R_{0}$, which is defined as the average out-degree of all non-seed nodes [18], for the three data sets are 0.246, 0.179, and 0.183, respectively. Accordingly, “Produce 101” demonstrates the highest transmissibility, followed by “Dying to survive”, while “Kunshan self-defense” exhibits the lowest transmissibility. All three data sets exhibit subcritical spreading dynamics—i.e., each node, on average, generates fewer than one offspring, leading inevitably to extinction over time.

### 3.2. Offspring Distributions

In a branching process, it is conventionally assumed that the offspring distribution—the probability $p_{k}$ that any given node produces exactly *k* offsprings—remains constant across all generations. This assumption implies that, across multiple independent diffusion trees governed by the same offspring distribution, the average empirical offspring distribution—defined as the probability ${\tilde{p}}_{k}$ that a node in any generation produces *k* offsprings for all trees—converges to and coincides with *p_k_*.

**Figure 2:**
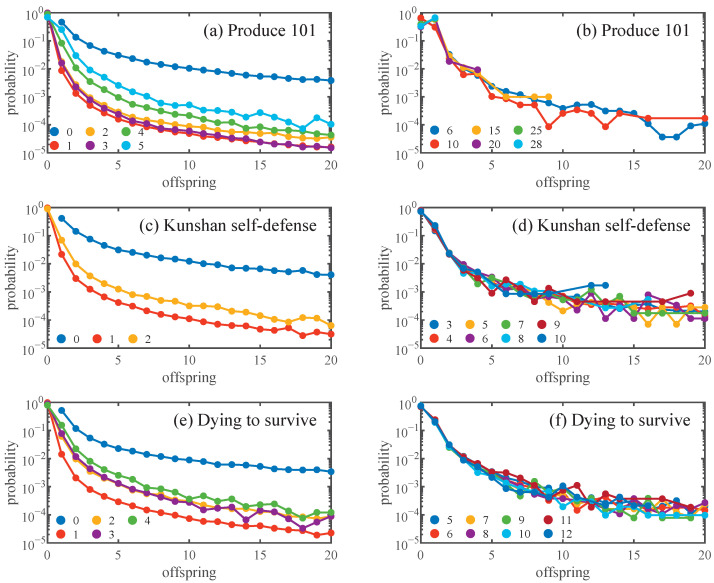
The average empirical offspring distribution per generation for the three data sets. (**a**,**c**,**e**) are for the generations before the stable ones, while (**b**,**d**,**f**) are for the stable generations. For (**b**), due to the large number of stable generations, offspring distribution for sample generations with approximately equal intervals is presented.

Figure 2 illustrates the average empirical offspring distribution per generation for the three data sets, restricted to diffusion trees with cascade sizes exceeding 1. Figure 2a,c,e reveals a marked distinction between the seed generation and subsequent ones: nodes in the seed generation are more likely to be forwarded and the probability of generating offsprings is greater. Furthermore, for cascades in the early generations, inter-generational variation in average offspring distribution can be more pronounced. According to Figure 1b,d,f, for generations exhibiting relatively stable effective branching numbers, the average offspring distribution is presented in Figure 2b,d,f. For all three data sets, the average empirical offspring distributions within the corresponding stable generations are found to be approximately consistent.

Theoretically, for a branching process characterized by specific parameters, the effective branching number should remain stable as the generation increases, and the average offspring distribution across generations in the diffusion trees should also be the same. According to the above empirical analysis, if the seed and non-seed generations are considered separately, all three data sets basically satisfy the assumptions of branching processes, thereby supporting its validity to model information diffusion. Moreover, diffusion cascades often exhibit a tree-like structure [18], providing an empirical basis for modeling spreading via branching processes.

**Figure 3:**
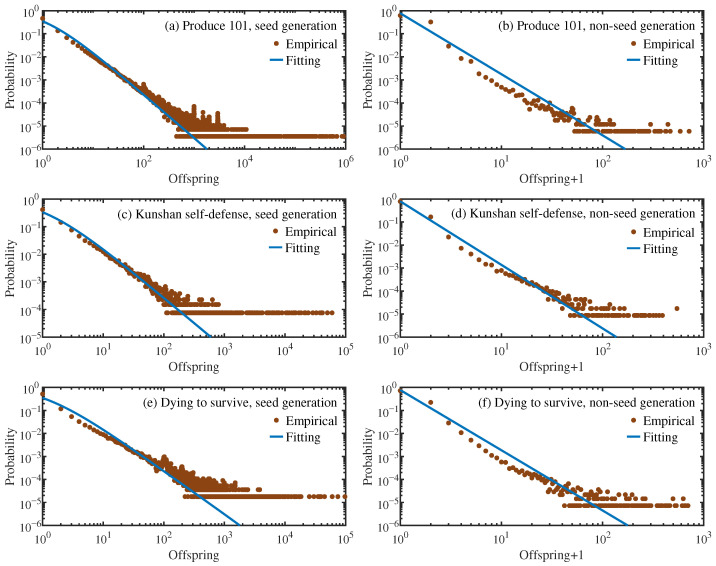
The offspring distributions of the seed (**a**,**c**,**e**) and stable non-seed (**b**,**d**,**f**) generations for the three data sets, along with the corresponding fitting lines.

Due to the significant differences in the offspring distribution of the seed generation compared to other generations, they are distinguished when fitting. For non-seed generations, the generations with stable offspring distribution for each data set are selected for fitting. Figure 3 presents the empirical offspring distributions. The seed generation shows a markedly right-skewed and heavy-tailed distribution, reflecting its stronger dissemination capabilities to generate a substantially larger number of forwardings. In contrast, the offspring distributions of the non-seed generations generally concentrate at lower offspring counts and decay more rapidly in the tail.

The offspring distribution exhibits an approximately linear decay on double-logarithmic axes; therefore, without loss of generality, this paper uses exponentially truncated power-law distributions to fit the data, assuming the offspring distribution follows

(1) $$p_{k}\propto {\left(k+1\right)}^{-\beta }e^{-\frac{k}{\theta }}.$$

Figure 3 presents the fitting results. The estimated parameters are summarized in Table 2.

Notably, the exponent β is significantly larger for non-seed generations than for seed generations, indicating a faster decay in the distribution tail in the former. Moreover, both generation types exhibit large θ values, suggesting that β plays a dominant role in the offspring distribution for most *k*, and θ takes effect when the offspring number *k* is large.

The confidence intervals for θ are consistently wide, which stems from the heavy-tailed nature of offspring number distribution: only a small fraction of nodes exhibit exceptionally large offspring counts. During bootstrap resampling, whether these nodes can be selected will significantly affect the tail of the distribution, thereby causing θ, which controls the exponential truncation, to fluctuate significantly between different samples, resulting in a larger confidence interval range. In contrast, the power-law exponent β is mainly determined by the offspring numbers of small and medium scales; as a result, β estimates exhibit greater robustness and narrower confidence intervals. If we remove a small number of nodes with large offspring numbers, we can find that it has little impact on the estimated β and its confidence interval, yet it markedly alters the estimated θ and reduces the width of its confidence interval. The MSEs across all three data sets are consistently low, indicating good fitting quality of the estimated distributions.

## 4. Branching Process Modeling

For the cascade size distribution of the three data sets, using the Kolmogorov–Smirnov goodness-of-fit statistical test, it is found that none of them pass the test with a threshold of p=0.1, indicating that the power-law distribution is not a reasonable model [52]. Several empirical studies show the critical behavior of information spreading: cascade sizes follow power-law distribution with exponent −3/2, which is consistent with a simple branching process with the mean μ=1 for offspring distribution [15,53,54]. However, as analyzed above, the data sets studied are not in the critical state.

We employ generating functions to analyze the cascade size distribution. Let ${\tilde{G}}_{\tau }\left(x\right)$ denote the generating function of the distribution of total node number from the 0th to the *τ*th generation (τ≥1) in the diffusion trees, $\tilde{f}\left(x\right)$ represent the generating function of the offspring distribution of seed nodes, and f(x) denote that of non-seed nodes. Then,

(2) $${\tilde{G}}_{\tau }\left(x\right)=x\tilde{f}\left(G_{\tau -1}\left(x\right)\right),$$

where $G_{\tau -1}\left(x\right)$ is the generating function of the distribution of total node number from the 0th to the (*τ* − 1)th generation for the subtrees rooted at the 1st generation nodes in the original trees, and it satisfies the recurrence relation

(3) $$G_{n}\left(x\right)=xf\left({G_{n}}_{-1}\left(x\right)\right),\;1\leq n\leq \tau -1,$$

with initial condition $G_{0}\left(x\right)=x$.

We can obtain the fitted offspring distributions from empirical data and subsequently obtain the generating function ${\tilde{G}}_{\tau }\left(x\right)$, which uniquely determines the cascade size distribution $p_{s}$, i.e., the inverse transform of ${\tilde{G}}_{\tau }\left(x\right)$. For the general form of generating functions, when they are only known at discrete points (applicable to the scenario of information diffusion), their inverse can be obtained by the Cauchy integral formula

$${\tilde{G}}_{\tau }\left(a\right)=\frac{1}{2\pi i}\oint_{\partial D}\frac{{\tilde{G}}_{\tau }\left(x\right)}{x-a}dx,$$

where $D=\{x:|x-x_{0}|\leq R\}$ is a closed disk fully contained within the domain of definition of ${\tilde{G}}_{\tau }$ (the neighborhood of $x_{0}$), ∂D denotes its boundary, and *a* is an arbitrary point inside *D*. For the *n*th derivative of ${\tilde{G}}_{\tau }$ at *a*, the following expression holds:

$${\tilde{G}}_{\tau }^{\left(n\right)}\left(a\right)=\frac{n!}{2\pi i}\oint_{\partial D}\frac{{\tilde{G}}_{\tau }\left(x\right)}{{\left(x-a\right)}^{n+1}}dx.$$

Therefore, the cascade size distribution $p_{s}$ is given by the following integral

$$p_{s}=\frac{1}{s!}{\tilde{G}}_{\tau }^{\left(s\right)}\left(0\right)=\frac{1}{2\pi i}\oint_{C}\frac{{\tilde{G}}_{\tau }\left(x\right)}{x^{s+1}}dx,$$

where *C* is a circle on the complex plane.

Let $x=Re^{iu}$, where *R* must satisfy that ${\tilde{G}}_{\tau }$ is analytic on *D*. Then we obtain

$$p_{s}=\frac{1}{2\pi R^{s}}\int_{0}^{2\pi }{\tilde{G}}_{\tau }\left(Re^{iu}\right)e^{-isu}du.$$

For numerical computations, the integral is evaluated using the trapezoidal rule, yielding an approximation of the true distribution $p_{s}$ (s=0,1,⋯,N−1) [31,46]:

(4) $${\tilde{p}}_{s}\approx \frac{1}{NR^{s}}\sum_{m=0}^{N-1}{\tilde{G}}_{\tau }\left(Re^{2\pi im/N}\right)e^{-2\pi ims/N},\;{\tilde{p}}_{s+N}={\tilde{p}}_{s}.$$

In this paper, we set R=1.0. Thus, $x=e^{iu}$, and *x* lies on the unit circle in the complex plane.

For the average empirical offspring distribution Equation (1), which follows an exponentially truncated power-law form, the corresponding generating function is

(5) $$f\left(x\right)=\frac{Li_{\beta }\left(xe^{-1/\theta }\right)}{xLi_{\beta }\left(e^{-1/\theta }\right)},$$

where $Li_{\beta }$ is the polylogarithm function of order β (also known as Jonquière’s function). We impose the condition that each seed node in the empirical data sets has at least one offspring. That is, we restrict the domain to k≥1. In this case, the generating function for the seed nodes is

(6) $$\tilde{f}\left(x\right)=\frac{x-e^{1/\theta }Li_{\beta }\left(xe^{-1/\theta }\right)}{x-xe^{1/\theta }Li_{\beta }\left(e^{-1/\theta }\right)}.$$

Based on Equations (5) and (6) and the recurrence relation Equations (2) and (3), we can obtain ${\tilde{G}}_{\tau }\left(x\right)$, and utilizing Equation (4), we can obtain ${\tilde{p}}_{s}$ (for sufficiently large *τ*).

**Figure 4:**
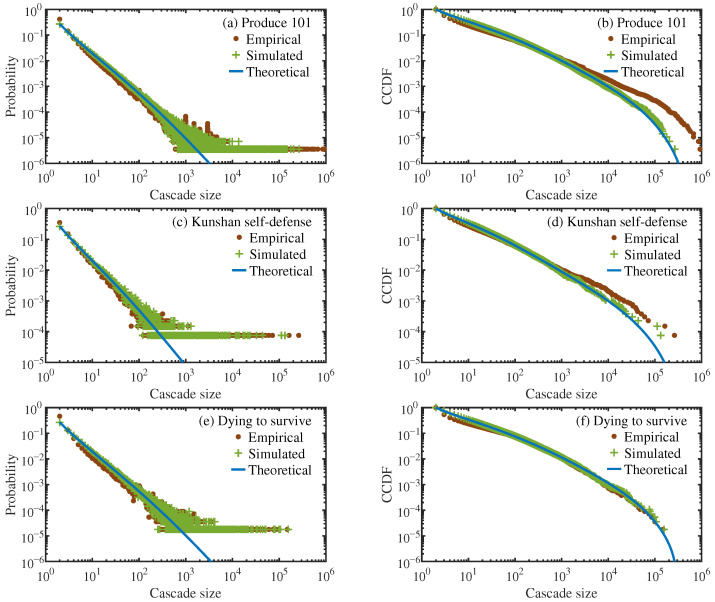
Comparison of cascade size distributions from empirical data, numerical simulations, and theoretical predictions using Equation (4) for three data sets. (**a**,**c**,**e**) are for probability distribution functions, while (**b**,**d**,**f**) are for complementary cumulative distribution functions (CCDFs).

The branching process can be simulated based on the fitted offspring distributions for seed and non-seed generations, as derived in the preceding section. The seed nodes are successively forwarded generation by generation until the diffusion terminates, and the simulation count is equal to the number of original posts that were once forwarded. Figure 4 presents three cascade size distributions—the empirical one from real data, the one obtained via numerical simulations, and the theoretical one predicted by Equation (4)—across three data sets. It can be seen that for both probability distributions and CCDFs, the simulation results and theoretical predictions are consistent, indicating the reliability of Equation (4), and both are also in good agreement with the cascade size distributions from real data.

When the cascade size is relatively small, there is a certain deviation between the real distribution and the theoretical prediction, which might be due to the noise in the real data and the finite-sample effect arising from limited observational data. After all, as shown in Figure 2, the average empirical offspring distributions across non-seed generations exhibit considerable variability.

At the distribution tail, the empirical distribution exhibits a distinct heavy-tailed behavior. The numerical simulation can capture this feature, while the theoretical prediction shows a certain degree of deviation in the large-cascade region, which is likely attributable to factors such as the finite-scale truncation and the finite-sample effects. Nevertheless, the overall agreement among theoretical predictions, numerical simulations, and empirical observations for meme popularity distribution verifies the validity of the branching process model for characterizing information diffusion on social media.

## 5. Discussion

As shown in Figure 1, the effective branching numbers across generations in social media are typically time-varying. The difference can be reflected in the variation of offspring distributions. This paper examines branching process modeling in which the offspring distribution differs between the seed generation and subsequent (non-seed) generations. In fact, assume that, for a given generation of nodes, the offspring distribution is identical across all nodes in that generation; then, the analytical framework developed in Section 4 remains applicable even when the offspring distribution varies across every generation. Let $f_{i}\left(x\right)$ denote the generating function of the offspring distribution of the *i*-th generation nodes (i≥0); according to Equations (2) and (3), we find that ${\tilde{G}}_{1}\left(x\right)=xf_{0}\left(x\right)$, ${\tilde{G}}_{2}\left(x\right)=xf_{0}\left(xf_{1}\left(x\right)\right)$, ${\tilde{G}}_{3}\left(x\right)=xf_{0}\left(xf_{1}\left(xf_{2}\left(x\right)\right)\right)$, ${\tilde{G}}_{4}\left(x\right)=xf_{0}\left(xf_{1}\left(xf_{2}\left(xf_{3}\left(x\right)\right)\right)\right)$, … Based on the recurrence relation, we can obtain ${\tilde{G}}_{\tau }\left(x\right)$.

Classical branching process theory has shown that when the offsprings follow a specific distribution and the mean offspring μ=1, cascade size distribution will follow a power law with exponent −3/2. While when μ<1, the distribution will approximately follow a power law with the same exponent but with an exponential cutoff. In both cases, the branching process will stop.

The previous section approximately obtains the cascade size distribution from a numerical computational perspective. For certain offspring distributions, the exact distributions of cascade sizes can be analytically obtained from their generating functions via the Lagrange inversion theorem, which has been applied to analyze the component size distribution for random graphs [55].

Assume that the offspring numbers of seed and non-seed nodes are independent and identically distributed (i.i.d.), with a common generating function f(x). Applying the Lagrange inversion theorem, the cascade size distribution $p_{s}$ can be expressed as

$$p_{s}=\frac{1}{s!}{\left[\frac{d^{s-1}}{dx^{s-1}}{\left(f(x)\right)}^{s}\right]}_{x=0}=\frac{1}{s}\left[x^{s-1}\right]{\left(f\left(x\right)\right)}^{s},\;s\geq 1,$$

where $\left[x^{s-1}\right]$ denotes the coefficient of $x^{s-1}$ term in the power series expansion of ${\left(f\left(x\right)\right)}^{s}$.

Specifically, assume the offspring distribution follows Poisson distribution $p_{k}=\frac{\lambda^{k}e^{-\lambda }}{k!}$ (λ>0, k≥0), and its generating function is

$$f\left(x\right)=\sum_{k=0}^{\infty }p_{k}x^{k}=\sum_{k=0}^{\infty }\frac{\lambda^{k}e^{-\lambda }}{k!}x^{k}=e^{-\lambda }\sum_{k=0}^{\infty }\frac{{\left(\lambda x\right)}^{k}}{k!}=e^{\lambda (x-1)}.$$

According to Lagrange inversion theorem, we obtain

$$p_{s}=\frac{{\left(s\lambda \right)}^{s-1}e^{-s\lambda }}{s!}.$$

For a sufficiently large *s*, Stirling’s approximation yields

$$p_{s}\approx \frac{1}{\lambda e\sqrt{2\pi }}s^{-\frac{3}{2}}e^{-(\lambda -\ln\lambda -1)s}.$$

When λ>0, (λ−lnλ−1)≥0, with equality attained if λ=1. This special case demonstrates that, when the mean offspring 0<μ<1, the size distribution of large cascades asymptotically follows an exponentially truncated power-law distribution with exponent −3/2. When λ=1, i.e., at the critical point,

$$p_{s}\approx \frac{1}{e\sqrt{2\pi }}s^{-\frac{3}{2}}\propto s^{-\frac{3}{2}},$$

indicating a power-law form with exponent −3/2.

Assume the offspring distribution follows exponential distribution $p_{k}=\left(1-e^{-\lambda }\right)e^{-\lambda k}$ (λ>0,k≥0), and its generating function is

$$f\left(x\right)=\frac{e^{\lambda }-1}{e^{\lambda }-x}.$$

By applying Lagrange inversion theorem and Stirling’s approximation, we obtain

$$p_{s}\approx \frac{e^{\lambda +1}}{4\sqrt{\pi }}s^{-\frac{3}{2}}e^{-s\ln\left[e^{2\lambda }{\left(e^{\lambda }-1\right)}^{-1}/4\right]}.$$

When λ>0, $\ln\left[e^{2\lambda }{\left(e^{\lambda }-1\right)}^{-1}/4\right]\geq 0$, with equality attained if $e^{\lambda }=2$ (in this case the mean offspring $\mu =1/(e^{\lambda }-1)=1$). When the mean offspring 0<μ<1, the size distribution of large cascades also asymptotically follows an exponentially truncated power-law distribution with exponent −3/2. When μ=1,

$$p_{s}\approx \frac{e}{2\sqrt{\pi }}s^{-\frac{3}{2}}\propto s^{-\frac{3}{2}},$$

also indicating a power-law form with exponent −3/2.

## 6. Conclusions

In this study, we add evidence for the branching process model proposed by Gleeson et al. [23] for information diffusion in social media. By calibrating model parameters against empirical data, the model can replicate the cascade size distributions observed in data well, and the theoretical predictions are in good agreement with empirical results. The study validates, both empirically and theoretically, the applicability of branching processes in information diffusion research.

This study adds to the existing body of data-driven models for information spreading. However, as revealed by real data, effective branching numbers and offspring distributions do not always remain constant, resulting in systematic deviations between theoretical predictions and empirical observations. During information dissemination, the basic reproductive numbers or effective branching numbers can be time-varying [56]. Variations in offspring distribution occur both across generations and within individual generations. In addition, due to information interaction, the diffusion of a piece of information may be either inhibited or amplified by the concurrent diffusion of other information [16].

Moreover, users exhibit heterogeneous influence in information diffusion on social media. The posts by super users can be shared by more people; thus, the node influence parameter or a multitype branching process can be utilized to study the information diffusion considering users with different types. This constitutes a promising avenue for future research.

The temporal data associated with users’ posts in our data sets is incomplete. Were the timestamps fully available, with these temporal characteristics taken into consideration, the discrete time branching model may be modified to a continuous-time branching process or an age-dependent one if the inter-generation time-interval distribution is not exponentially distributed.

In social media, users typically retweet posts published or previously retweeted by accounts they follow. Previous studies have shown that the dynamical patterns of the diffusion process are strongly conditioned by the topology of the underlying network of followers [57]. The appearance of a small fraction of extremely efficient users results from the heterogeneity of the underlying network and independently of the individual user behavior. However, our data sets do not include user-to-user following relationships; consequently, reconstructing a follower–followee network is infeasible.

Additionally, we are also unable to obtain several important attributes of users, such as the number of their followers and followees, as well as the number of posts shown to them. Thus, we cannot study the impact of saturation effect on information diffusion shown in previous studies [13,58]. The effect occurs due to limitations in user attention, cognitive processing capacity, and the natural lifecycle of content. At the user level, when users follow a large number of accounts, they are exposed to an overwhelming volume of content, which can reduce their likelihood of retweeting posts [13]. At the macro level, information can spread through distinct phases on social media—expansion, front-page, and saturation [58]. The final phase occurs when information ages and its rate of spread slows down significantly. Future works should integrate these aspects, and propose more elaborate, data-driven models of information diffusion to address the complexity of real-world scenarios.

## Figures and Tables

**Table 1:** The numbers of all posts, original posts, reposts, and reposted among the original posts that were subsequently forwarded by other users for the three data sets.

Count	Produce 101	Kunshan Self-Defense	Dying to Survive
All posts	89,789,623	1,738,633	4,775,568
Original posts	33,522,289	76,837	786,602
Reposts	56,267,334	1,661,796	3,988,966
Reposted among the original	281,373	13,270	55,958

**Table 2:** Fitting results for the offspring distributions of the seed and stable non-seed generations. The 95% confidence intervals (CIs) obtained by bootstrapping and mean squared errors (MSEs) are also presented. For bootstrapping, from the original data of the offspring numbers of nodes, we draw 10^3^ random samples consisting of the same number of nodes uniformly with replacement. For each sample, we obtain *β* and *θ*. The 95% confidence intervals are computed using the 2.5 and 97.5% percentiles of the 10^3^ samples. Given the data characteristics and fitting performance, maximum likelihood estimation is adopted for seed generations, and moment estimation is applied for non-seed generations.

Parameter	Produce 101, Seed	Kunshan Self-Defense, Seed	Dying to Survive, Seed	Produce 101, Non-Seed	Kunshan Self-Defense, Non-Seed	Dying to Survive, Non-Seed
β	1.872	1.825	1.869	2.640	2.761	2.617
β’s CI	[1.867, 1.878]	[1.809, 1.841]	[1.860, 1.880]	[2.621, 2.659]	[2.730, 2.790]	[2.586, 2.648]
θ	41,637.483	54,429.579	34,353.213	4422.131	28,089.851	26,404.088
θ’s CI	[34,847.003, 165,831.909]	[23,346.038, 334,022.358]	[18,352.977, 133,404.814]	[2369.704, 7246.580]	[11,528.280, 132,694.614]	[16,802.148, 42,208.411]
MSE	$5.663\times 10^{-6}$	$1.547\times 10^{-5}$	$2.753\times 10^{-5}$	$4.010\times 10^{-4}$	$2.149\times 10^{-5}$	$6.934\times 10^{-5}$

## Data Availability

The data sets supporting the conclusions of this paper are available at https://doi.org/10.6084/m9.figshare.31914564.
